# Carbon Dots and Biomimetic Membrane Systems: Mechanistic Interactions and Hybrid Nano-Lipid Platforms

**DOI:** 10.3390/nano16020140

**Published:** 2026-01-20

**Authors:** Nisreen Nusair, Mithun Bhowmick

**Affiliations:** Department of Mathematical and Physical Sciences, Miami University, 4200 N. University Blvd., Middletown, OH 45042, USA; bhowmim@miamioh.edu

**Keywords:** Carbon Dots (CDs), nanomaterials, model membrane system, liposomes, vesicles, interactions, hybrid, biomedical applications, physicochemical, drug delivery

## Abstract

Carbon dots (CDs) have emerged as a distinct class of fluorescent nanomaterials distinguished by their tunable physicochemical properties, ultrasmall size, exceptional photoluminescence, versatile surface chemistry, high biocompatibility, and chemical stability, positioning them as promising candidates for biomedical applications ranging from sensing and imaging to drug delivery and theranostics. As CDs increasingly transition toward biological and clinical use, a fundamental understanding of their interactions with biological membranes becomes essential, as cellular membranes govern nanoparticle uptake, intracellular transport, and therapeutic performance. Model membrane systems, such as phospholipid vesicles and liposomes, offer controllable platforms to elucidate CD-membrane interactions by isolating key physicochemical variables otherwise obscured in complex biological environments. Recent studies demonstrate that CD surface chemistry, charge, heteroatom doping, size, and hydrophobicity, together with membrane composition, packing density, and phase behavior, dictate nanoparticle adsorption, insertion, diffusion, and membrane perturbation. In addition, CD-liposome hybrid systems have gained momentum as multifunctional nanoplatforms that couple the fluorescence and traceability of CDs with the encapsulation capacity and biocompatibility of lipid vesicles, enabling imaging-guided drug delivery and responsive theranostic systems. This review consolidates current insights into the mechanistic principles governing CD interactions with model membranes and highlights advances in CD-liposome hybrid nanostructures. By bridging fundamental nanoscale interactions with translational nanomedicine strategies, this work provides a framework for the rational design of next-generation CD-based biointerfaces with optimized structural, optical, and biological performance.

## 1. Introduction

Carbon dots (CDs) have established themselves as a transformative class of fluorescent nanomaterials, defined by their ultrasmall size (<10 nm), tunable surface chemistry, robust photoluminescence, and excellent aqueous dispersibility [1,2,3,4]. Unlike traditional semiconductor quantum dots, CDs offer a carbon-based, environmentally friendly composition that confers superior biocompatibility and chemical versatility. These intrinsic attributes have positioned CDs as premier candidates for a broad spectrum of biomedical applications, ranging from bioimaging and biosensing to targeted drug delivery, gene therapy, and cancer theranostics [1,2,3,4].

Despite extensive development of CDs for biomedical use, a critical unresolved challenge remains: the lack of a mechanistic framework describing how CD physicochemical properties govern their interactions with lipid membranes. The cellular membrane serves as the primary barrier governing nanoparticle internalization, intracellular transport, and therapeutic efficacy. Yet, the complexity of native biological membranes, characterized by dynamic phase heterogeneity, diverse protein content, and active signaling pathways, often obscures the fundamental physicochemical mechanisms driving nanoparticle engagement. Understanding these interactions is not merely academic; it is a safety imperative. Mechanisms of toxicity, such as membrane disruption or oxidative stress responses, are frequently initiated at this interface, making the elucidation of CD-membrane behavior a prerequisite for safe translational use [5,6,7,8,9,10,11,12].

To overcome the limitations of complex in vivo models, model membrane systems, specifically phospholipid vesicles and liposomes, have emerged as essential tools. These biomimetic platforms offer structural simplicity and tunable compositions, enabling researchers to isolate and systematically evaluate how variables such as lipid packing density, phase behavior, and fluidity influence CD adsorption and insertion [8,9,13,14]. Recent biophysical and computational studies utilizing these systems have begun to reveal governing principles. For instance, hydrophobic and positively charged CDs tend to penetrate lipid bilayers, whereas neutral or highly hydrated variants often remain surface-bound. Furthermore, advanced analytical approaches, including Molecular Dynamics (MD) simulations and Förster Resonance Energy Transfer (FRET), are now providing unprecedented insight into the nanoscale organization and dynamic remodeling of membranes upon CD contact [15,16,17,18].

Beyond serving as passive subjects of study, these membrane interactions are being actively engineered. The integration of CDs into lipid-based carriers has given rise to hybrid CD-liposome nanoplatforms that synergize the optical traceability of CDs with the encapsulation capacity and biocompatibility of lipid vesicles. These hybrid assemblies represent a new frontier in nanomedicine, offering enhanced stability and “all-in-one” capabilities for imaging-guided drug delivery and responsive theranostics.

This review consolidates current insights into two interconnected themes: (i) the interactions between CDs and biomimetic or model membrane systems, and (ii) the development and behavior of hybrid CD-lipid assemblies that incorporate CDs into lipid-based structures such as vesicles and liposomes. Section 2 and Section 3 in this review article provide the necessary foundational overview of CD properties and model membrane platforms; although these topics have been extensively documented in prior review articles, they are summarized here to establish essential context and maintain focus on the two central themes explored in this work.

Building on this foundation, the first major theme examines how membrane properties, including fluidity, rigidity, phase state, packing density, and the overall organization of bilayers that mimic biological membranes, together with key CD attributes such as surface chemistry, charge, hydrophobicity, size, and other physicochemical features, collectively dictate adsorption, insertion, diffusion, and membrane perturbation through electrostatic, hydrogen-bonding, and van der Waals interactions. These coupled factors determine the nanoscale structural remodeling, morphology, and dynamic behavior of the lipid bilayer upon CD engagement [6,7,8,9,13,14,19,20]. The second theme explores hybrid CD-lipid or CD-liposome systems in which CDs are embedded within or associated with lipid vesicles to form multifunctional nanoplatforms that combine the photoluminescence, fluorescence, and surface reactivity of CDs with the biocompatibility, encapsulation capacity, and structural tunability of lipid carriers. Such hybrids exhibit enhanced photoluminescence, strong interfacial coupling, and tunable physicochemical and biological stability, enabling imaging-guided drug delivery, responsive release, integrated theranostics, and broader biomedical applications [15,16,17,18].

Together, these perspectives provide critical insight into both the fundamental biophysical mechanisms governing CD-membrane interactions and the functional design principles underpinning hybrid CD-lipid nanostructures. By integrating computational, biophysical, and translational findings, this review aims to guide the development of next-generation carbon-based nanomaterials optimized for controlled membrane engagement, enhanced optical performance, and safe application in bioimaging, biosensing, phototherapy, drug delivery, and cancer theranostics.

## 2. Carbon Dots (CDs)

### 2.1. Classification, Characterization, and Properties of CDs

CDs have emerged as a versatile class of zero-dimensional carbon-based nanomaterials typically less than 10 nm in diameter, distinguished by their tunable optical, electronic, and chemical properties [21,22,23,24]. Their hybridized carbon cores, composed of mixed sp^2^/sp^3^ domains, combined with abundant surface functional groups, afford high surface area, excellent aqueous dispersibility, and diverse reactivity [25]. These features underpin their exceptional photoluminescence, high chemical and photostability, biocompatibility, and cost-effective synthesis, making CDs highly promising for biomedical applications, including drug, gene, and vaccine delivery, bioimaging, biosensing, theranostics, and antimicrobial or anticancer functions [21,22,24,26,27].

**Figure 1:**
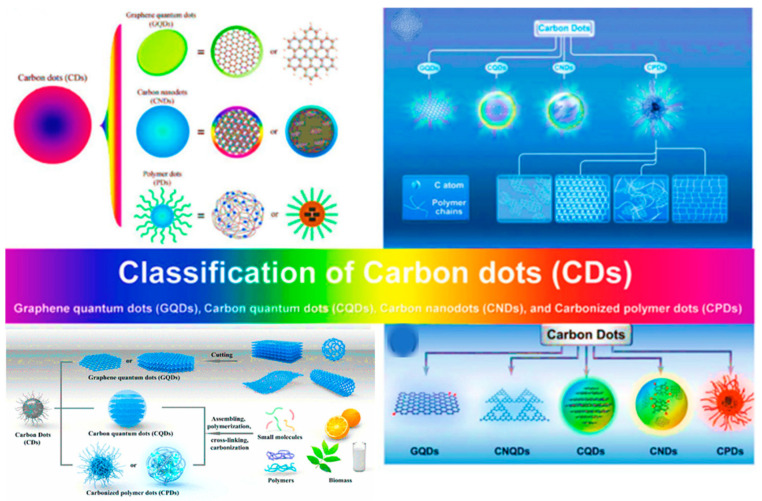
Classification of CDs. Reprinted with permission from Ref. [21].

CDs are generally classified into four major categories based on structural features, properties, and synthetic routes: graphene quantum dots (GQDs), carbon quantum dots (CQDs), carbon nanodots (CNDs), and carbonized polymer dots (CPDs), as illustrated in Figure 1 [21,24,28,29,30,31]. GQDs are nanoscale graphene fragments with predominantly sp^2^-hybridized, crystalline carbon structures whose photoluminescence arises from quantum confinement and edge effects. CQDs are quasi-spherical nanoparticles composed of mixed sp^2^/sp^3^ carbon domains, with fluorescence governed by both core size effects and surface states. CNDs are largely amorphous carbon nanoparticles in which emission originates mainly from surface defects and functional groups rather than quantum confinement. CPDs are partially carbonized polymer-derived nanoparticles whose fluorescence is dominated by molecular and polymeric surface states, enabling high tunability and strong emission. These types are well documented in the literature and differ in their degree of crystallinity, core composition, and surface states. Their varied structures impart distinct photophysical behavior, biocompatibility profiles, and suitability for different biomedical and technological applications [24,29].

The performance and biological behavior of CDs are dictated by several intrinsic properties, including particle size, hybridization structure, surface charge, solubility, and the nature of surface functional groups such as carboxyl, hydroxyl, and amine moieties [21,25,30]. Heteroatom doping (e.g., N, S, P) further tunes band structure, enhances quantum yield, and modulates catalytic or biological activity [21,29,31]. These attributes collectively affect their cell membrane interactions, cellular uptake, colloidal stability, and optical responses.

CDs exhibit strong UV absorption due to π–π* transitions of C=C bonds and n–π* transitions of C=O functionalities, with absorption tails extending into the visible region [21,22,29,32]. Their fluorescence often demonstrates excitation-dependent behavior, arising from multiple emissive centers, surface defects, and quantum confinement effects. Size-dependent emission enables tunability from visible to near-infrared wavelengths, facilitating applications in deep-tissue imaging and long-term in vivo tracking [29]. Surface passivation and heteroatom doping significantly enhance photoluminescence quantum yield, while environmental parameters such as pH, temperature, and ionic concentration influence emission intensity and stability [24,25,26,31,32,33].

Fluorescent CDs exhibit excellent biocompatibility, chemical inertness, nontoxicity, and environmental safety, distinguishing them from metal-based quantum dots that contain toxic heavy elements [22,25,28]. Their high photostability, aqueous solubility, and long-term chemical stability enable safe biological use and durable signal retention [21,22,26,34]. Notably, some CDs have demonstrated intrinsic antibacterial and anticancer activities, further expanding their biomedical potential [24,27,32].

In addition to experimental characterization, computational studies have begun to provide valuable molecular-level insights that complement and refine our understanding of how CD structure and surface chemistry influence stability and function. In particular, recent MD simulations have provided deeper insight into how structural features and surface chemistry govern CD stability and internal dynamics in aqueous environments [35]. CDs approximately 2 nm in diameter remained structurally compact when their surfaces were enriched in hydrogen-bonding functional groups, such as uncharged carboxyl, hydroxyl, and carbonyl moieties. These groups formed extensive intermolecular hydrogen-bond networks that reinforced the multilayered carbon core. In contrast, negatively charged CDs bearing ionized carboxyl groups displayed reduced cohesion and a tendency to exfoliate into smaller fragments, underscoring the sensitivity of CD integrity to surface charge state. The simulations also revealed internal rotational mobility of carbon layers, with rotation rates decreasing as particle size and surface functionalization increased. Functional groups restricted motion most strongly in the central layers, while outer layers remained comparatively dynamic. These computational findings complement experimental observations by illustrating how surface chemistry, charge neutralization, and functional group density contribute not only to colloidal stability but also to the internal structural organization and flexibility of CDs, parameters that ultimately influence photophysical behavior, biological interactions, and environmental responsiveness [35].

Overall, CDs offer a unique combination of structural tunability, strong and tunable photoluminescence, biocompatibility, and chemical versatility. Rigorous characterization remains foundational to advancing CD science, enabling the precise tuning of physicochemical and optical properties for targeted applications in bioimaging, sensing, drug and gene delivery, catalysis, environmental monitoring, and beyond [21,24,28,29].

### 2.2. CD Synthesis Approaches

The synthesis of CDs is generally categorized into bottom-up and top-down approaches [21,22,23,24,25,26,28,29,34]. Both methods aim to produce CDs with controlled size, morphology, surface chemistry, and optical properties, which are crucial for their performance in various applications such as biological imaging, biosensing, and optoelectronics. These synthesis methods have been extensively studied and reviewed in the literature, reflecting their central importance in the development of carbon-based nanomaterials. Despite the convenience of CD production, several challenges persist, including nanoparticle aggregation during carbonization, achieving uniform size and shape, and optimizing surface functionalization. Moreover, the chosen synthesis route significantly influences the physicochemical characteristics of CDs, including their structural, absorption, fluorescence, and overall optical properties, thereby determining their suitability for specific applications [21].

In the top-down approach, CDs are directly synthesized by breaking down bulk carbon materials [28]. These techniques encompass laser ablation, arc discharge, chemical oxidation, ball milling, combustion, plasma breakdown, chemical exfoliation, ultrasonic-assisted treatment, sono-chemical breakdown, and the electrochemical method [21,26]. Such methods rely on the fragmentation or exfoliation of larger carbonaceous precursors, such as graphene oxide, graphite, carbon nanotubes, and carbon fibers, into nanoscale particles. Top-down synthesis is generally scalable and cost-effective, utilizing readily available carbon sources. However, it provides limited control over surface chemistry, functionalization, and size uniformity compared to bottom-up methods [21,24,25,29,34].

Conversely, the bottom-up approach involves the construction of CDs from small molecular precursors through controlled chemical reactions [28]. This approach includes solvothermal synthesis, microwave-assisted synthesis, sono-chemical synthesis, thermal decomposition, reverse micelle synthesis, solid-state reactions, electro-oxidation, pyrolysis/carbonization, hydrothermal method, and chemical vapor deposition [21,26]. These methods rely on carbonization, surface passivation, size control, and purification processes to build up the carbon nanostructure from molecular components. Bottom-up synthesis provides precise control over particle size, surface functionalities, and optical properties, allowing for the fine-tuning of CDs for targeted applications. Nevertheless, it can be time-consuming and requires meticulous optimization of reaction conditions and purification steps to ensure uniformity and reproducibility [21,22,24,25,28,29,34].

The selection of a synthesis strategy depends on the desired physicochemical properties, production scale, and intended application of the CDs. In many cases, researchers employ hybrid or combined approaches to leverage the advantages of both methods, thereby enhancing synthesis efficiency and tailoring CD characteristics for specific functional applications [21].

### 2.3. Biomedical Applications of CDs

CDs have emerged as highly versatile nanomaterials in biomedical research due to their exceptional photostability, strong photoluminescence, chemical inertness, and excellent dispersibility in both aqueous and organic media [21,24]. Their low cytotoxicity, superior biocompatibility, tunable surface functionality, and facile synthesis have drawn significant attention in healthcare and nanomedicine [23]. These attributes enable CDs to function as multifunctional agents across a broad range of applications, including bioimaging, biosensing, drug, vaccine, and gene delivery, phototherapy, and other therapeutic modalities [23]. Their ability to integrate both diagnostic and therapeutic capabilities within a single nanoplatform positions CDs as ideal candidates for cancer theranostics, an emerging approach that combines diagnosis and therapy, to improve treatment precision and monitoring [36].

#### 2.3.1. Drug, Vaccine, and Gene Therapy

Among biomedical applications, drug delivery represents one of the most advanced and promising uses of CDs, especially within the context of theranostics [21,23,24,31,33]. Their ultrasmall size, typically less than 10 nm, allows them to penetrate biological membranes and traverse physiological barriers such as the blood–brain barrier (BBB). The surface of CDs contains abundant oxygen- and nitrogen-containing functional groups (carboxyl, hydroxyl, and amine), which facilitate drug conjugation, ligand attachment, and controlled release. Their intrinsic fluorescence provides real-time tracking of drug biodistribution and release, integrating diagnostic and therapeutic functions within a single platform [29,37].

In cancer therapy, CDs have been widely used as carriers for chemotherapeutic agents such as doxorubicin, 5-fluorouracil, methotrexate, and curcumin [23,38,39,40]. Drug conjugation to CDs enhances solubility, stability, and bioavailability, while reducing nonspecific toxicity. Many CD-drug hybrid systems exhibit pH-dependent release behavior, enabling accelerated release under the acidic tumor microenvironment (pH ≈ 5), thereby increasing tumor specificity. Functionalization with targeting ligands such as folic acid, hyaluronic acid, or transferrin promotes receptor-mediated uptake and selective accumulation in tumor tissues [41,42,43,44,45,46,47]. The combination of CD-based drug delivery with photodynamic or photothermal therapy allows for synergistic effects, where light activation enhances both reactive oxygen species (ROS) generation and drug release [41,45].

Beyond cancer therapy, CDs are being explored for antimicrobial drug delivery, where they can act as both carriers and active antibacterial agents [21,23,48]. Their cationic surface facilitates electrostatic interactions with negatively charged bacterial membranes, leading to membrane disruption, cytoplasmic leakage, and ROS-mediated oxidative stress. Under light irradiation, CDs can generate ROS that damage bacterial DNA, proteins, and lipids, making them promising agents against antimicrobial-resistant strains. CDs can also inhibit biofilm formation and enhance the efficacy of conventional antibiotics [23,49].

In ocular drug delivery, CDs improve corneal permeability, reduce irritation, and extend drug residence time when incorporated into polymeric gels or composites. Their fluorescence enables simultaneous tracking of drug delivery and distribution within ocular tissues [50]. For brain-targeted delivery, CDs are particularly attractive due to their ability to cross the BBB via passive diffusion, adsorptive transcytosis, or receptor-mediated endocytosis [38]. Ligand-functionalized or amino acid-derived CDs have been used to deliver chemotherapeutics to glioma cells or neuroprotective agents to neuronal tissues, offering promising results in treating neurological disorders [23,51].

In gene delivery, CDs act as efficient, low-toxicity non-viral vectors for transferring genetic material such as siRNA and plasmid DNA. Their positively charged surfaces promote strong binding with nucleic acids, resulting in compact, stable complexes with high transfection efficiency. Moreover, the inherent fluorescence of CDs allows visualization of gene transport and expression within cells, facilitating imaging-guided gene therapy [47]. CDs have additionally been explored as vaccine nanocarriers and adjuvants due to their ability to activate immune cells and stimulate Th1-biased immune responses [23,52].

Recent advances also focus on stimuli-responsive and controlled-release systems, where CDs are engineered to release drugs in response to specific triggers such as pH, temperature, redox potential, or enzymatic activity. Hollow CDs and CD-hydroxyapatite composites, for example, exhibit sustained and pH-responsive drug release ideal for tumor environments [23,53]. These multifunctional and traceable systems demonstrate the immense potential of CDs as intelligent nanocarriers in advanced theranostic applications.

#### 2.3.2. Bioimaging

CDs have gained considerable interest as next-generation fluorescent probes due to their high quantum yield, excellent photostability, and low toxicity compared to conventional dyes and semiconductor quantum dots [22,24,25,29,32]. Their fluorescence emission, which can be excitation-dependent or independent, enables multicolor imaging of biological structures with high resolution in both in vitro and in vivo systems. CDs can be internalized through endocytosis or passive diffusion and used for organelle-specific imaging, including the nucleus, lysosomes, and mitochondria.

In vivo, red and near-infrared emissive CDs have proven particularly useful for deep tissue imaging because they reduce background autofluorescence and light scattering [32,38,54]. Certain carbonized polymer dots (CPDs) have shown the ability to cross the BBB without the need for targeting ligands, offering potential for neurological imaging and monitoring. CDs have also been integrated into multimodal imaging platforms, such as fluorescence combined with magnetic resonance or photoacoustic imaging, which enhances spatial resolution and diagnostic accuracy for theranostic applications [31,32,54].

#### 2.3.3. Biosensing

The fluorescence of CDs is highly sensitive to environmental changes, making them excellent candidates for biosensing and clinical diagnostics [22,25,29,30,32,55,56]. Their sensing mechanisms typically rely on fluorescence quenching, enhancement, or wavelength shifts caused by interactions with analytes, allowing for quantitative and selective detection [38]. CDs have been successfully employed to detect metal ions such as Fe^3+^, Cu^2+^, Hg^2+^, and Pb^2+^, which are important in monitoring degenerative and metabolic disorders [1,29,33]. They have also been used for detecting biomolecules, including ATP, dopamine, ascorbic acid, DNA, and siRNA, as well as biomarkers such as Nuclear Matrix Protein 22 for bladder cancer diagnosis [23,57,58,59,60,61].

Functionalization of CDs with recognition elements such as enzymes, antibodies, or aptamers enhances their selectivity and expands their clinical utility. Furthermore, CDs are effective for detecting bacterial pathogens, including Staphylococcus aureus and Escherichia coli, and for monitoring environmental changes such as pH and temperature in living cells [32,34,62,63]. Their stability, biocompatibility, and tunable optical response make them ideal for real-time diagnostic and monitoring systems in nanomedicine.

#### 2.3.4. Nanomedicine and Therapeutic Activity

CDs are rapidly evolving to serve as active therapeutic agents with direct antibacterial, antiviral, and anticancer properties [21,23]. When synthesized from pharmacologically active precursors such as curcumin or metronidazole, CDs often retain pharmacological effect while significantly improving solubility, fluorescence, and bioactivity compared to the original drugs [38,64]. CD-based systems are frequently paired with photodynamic therapy (PDT) or photothermal therapy (PTT). In these scenarios, light irradiation triggers the CDs to generate ROS or heat, destroying tumor cells or bacteria while simultaneously releasing the drug. Additionally, CDs can function as vaccine adjuvants by stimulating dendritic cell maturation and T-cell activation, while their fluorescence enables real-time tracking of antigen uptake and immune response [38].

In summary, the multifunctional behavior of CDs positions them as a new generation of “all-in-one” nanomaterials with broad biomedical potential [31]. Their unique combination of biocompatibility, tunable photophysical properties, and versatile surface chemistry enables simultaneous bioimaging, biosensing, and therapeutic applications within a single platform [23]. Among these, drug delivery remains the most extensively developed and promising field, offering image-guided, targeted, and stimuli-responsive therapy. By integrating diagnostic and therapeutic functions, CDs embody the core principles of theranostics and support the development of personalized, precise, and minimally invasive healthcare solutions. Continued investigation into their long-term biosafety and structure-function relationships will be key for advancing CDs toward establishing their role in next-generation nanomedicine.

## 3. The Model Membrane System (Phospholipid Vesicles or Liposomes)

**Figure 2:**
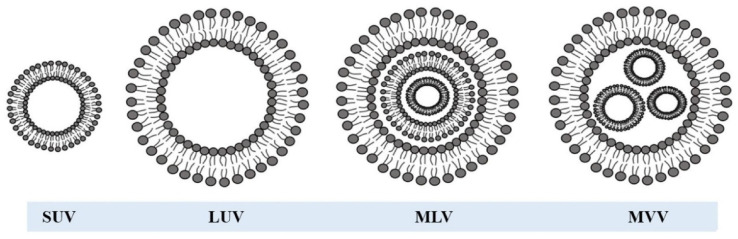
Classification of phospholipid vesicles or liposomes based on structure and size. SUV: small unilamellar vesicle (20–100 nm), LUV: large unilamellar vesicles (100–1000 nm), MLV: multilamellar vesicles (>500 nm), and MVV: multivesicular vesicles (>1000 nm). Reprinted with permission from Ref. [71].

Model membrane systems are simplified, biomimetic assemblies that replicate the fundamental structural and physicochemical properties of biological membranes, while eliminating the complexity and variability inherent in living systems [65,66,67,68,69]. They are primarily composed of phospholipid bilayers that self-assemble into vesicular structures, such as liposomes, when dispersed in an aqueous medium, as shown in Figure 2 [70,71]. These lipid bilayers exhibit the same amphiphilic organization as natural cell membranes, with hydrophilic headgroups facing the aqueous environment and hydrophobic tails forming the inner core. The amphiphilic nature of the lipids drives their spontaneous organization into stable bilayers, mimicking the selective permeability, fluidity, and compartmentalization observed in biological membranes [66,67,68,69]. Vesicles can vary in size and structure, ranging from small unilamellar vesicles (SUVs, 20–100 nm) and large unilamellar vesicles (LUVs, >100 nm) to multilamellar vesicles (MLVs), which contain multiple concentric bilayers [26,71]. Their composition can be finely tuned by varying the types of phospholipids and additives such as cholesterol, sphingolipids, or glycolipids, thereby modifying parameters like surface charge, rigidity, and permeability [9].

The physicochemical characteristics of these membranes, such as fluidity, surface potential, phase behavior, and permeability, depend on factors including lipid chain length, degree of unsaturation, temperature, and the presence of charged or neutral lipids [26]. For example, saturated lipids like dipalmitoyl phosphatidylcholine (DPPC) exhibit gel-to-fluid phase transitions near physiological temperatures, while unsaturated lipids like dioleoyl phosphatidylcholine (DOPC) form more fluid and permeable bilayers at room temperature. These tunable characteristics make model membranes ideal systems for studying how external molecules interact with lipid bilayers under controlled experimental conditions. They provide a reproducible environment for probing molecular-level phenomena such as adsorption, diffusion, penetration, and disruption of the membrane by drugs, peptides, or nanomaterials [8,72].

Model membrane systems are particularly valuable tools for investigating the interactions of nanomaterials with biological interfaces [26]. Their simplicity allows precise quantification of binding affinity, insertion depth, and induced changes in bilayer properties, such as fluidity or permeability. For instance, studies using liposomal models have revealed that nanoparticle charge, size, and surface chemistry strongly influence their affinity toward membranes. Positively charged nanoparticles tend to interact more strongly with negatively charged bilayers, promoting adsorption, whereas hydrophobic nanoparticles are often found embedded within the bilayer core, facilitating insertion. These insights are essential for understanding nanoparticle behavior in biological systems, including potential cytotoxicity, cellular uptake, and membrane perturbation [6,7,8,9,14,17,18,20,26].

Beyond serving as fundamental biophysical models, lipid vesicles are also practical carriers in drug delivery research [26]. Their bilayer structure enables encapsulation of both hydrophilic compounds within the aqueous core and hydrophobic molecules within the lipid region. Liposomes can therefore act as both model membranes and functional nanocarriers, allowing simultaneous studies of drug encapsulation, release kinetics, and permeability enhancement induced by interacting agents such as CDs. Hybrid systems that combine liposomes with CDs have been developed to explore nanocarrier stability, membrane permeability, and nanoparticle localization within the bilayer. These studies not only provide mechanistic insights into membrane–nanomaterial interactions but also guide the rational design of advanced nanocarriers for targeted drug delivery and theranostic applications [9,18,26].

Overall, model membrane systems represent an essential experimental framework for bridging the gap between molecular-scale studies and complex biological environments. Their structural simplicity, tunable composition, and biophysical relevance make them powerful platforms for elucidating the interaction mechanisms of drugs, peptides, and nanomaterials with cellular membranes. Through these systems, researchers can better predict nanoparticle behavior in vivo, optimize nanocarrier formulations, and develop safer, more effective biomedical applications.

## 4. Carbon Dots and the Model Membrane Systems

### 4.1. Interactions of the CDs with the Model Membrane System

The interaction of CDs with model membrane systems is governed by a complex interplay of physicochemical and structural factors that determine their behavior at lipid interfaces [26,73]. These interactions primarily depend on the surface chemistry of the CDs, which determines their charge distribution, hydrophobicity, and capacity for electrostatic, hydrogen bonding, and van der Waals interactions with lipid molecules. At the same time, the intrinsic properties of the membrane, such as fluidity, rigidity, molecular packing density, and phase state, define the extent of adsorption, insertion, diffusion, and permeability of CDs within the bilayer. When these parameters align favorably, CDs can integrate into or traverse the membrane, whereas unfavorable conditions lead to surface adsorption or aggregation. Understanding these interdependent factors is crucial for elucidating how CDs are influenced by membrane structure, dynamics, and function and for guiding the design of CDs in biomedical applications such as bioimaging, drug delivery, and biosensing [26].

#### 4.1.1. Molecular Dynamics Insights into Membrane-Density-Dependent CD Permeation

**Figure 3:**
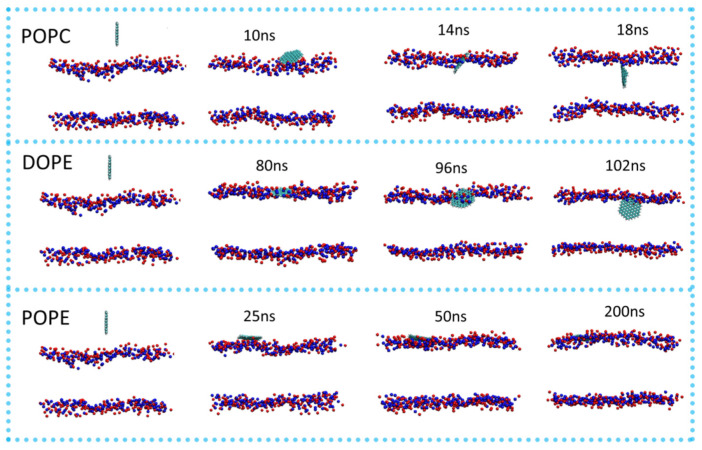
Permeation of CDs (green object) through different cell membranes. P atoms (blue dots) and N atoms (red dots) in the cell membrane. Reprinted with permission from Ref. [6].

MD simulations provide a mechanistic framework for understanding how membrane molecular density governs CD permeation across chemically distinct lipid bilayers. Figure 3 shows the transport mechanism of CDs across phospholipid membranes as analyzed utilizing MD simulations to probe the underlying factors that govern permeation and adsorption. The study specifically focused on GQDs, a type of CDs, with a radius of 0.85 nm and their interactions with three different model membranes composed of 1-palmitoyl-2-oleoyl-sn-glycero-3-phosphocholine (POPC), 1,2-dioleoyl-sn-glycero-3-phosphoethanolamine (DOPE), and 1-palmitoyl-2-oleoyl phosphatidylethanolamine (POPE). The results clearly demonstrated that the ability of CDs to permeate the membrane interior is highly dependent on the phospholipid molecular density of the membrane, which directly influences both the free energy landscape and the strength of interaction forces involved in translocation [6].

The MD simulations in Figure 3 revealed that CDs were able to permeate POPC and DOPE membranes easily. In the case of POPC, the CDs reached the membrane surface within approximately 10 ns and rapidly penetrated the bilayer. During this process, the CDs became embedded within the phospholipid molecules, maintaining an orientation parallel to the phospholipid tails and stably adhering within the interior of the membrane. Similarly, in the DOPE system, the CDs were initially absorbed by the membrane around 80 ns, after which they quickly permeated the bilayer. The CDs are inserted into the hydrophobic interior of the membrane, maintaining a parallel orientation relative to the phospholipid chains and achieving stable integration within the lipid matrix. These observations indicate that both POPC and DOPE membranes provide sufficient free space between lipid molecules to allow CDs to overcome energy barriers and embed into the bilayer without significant structural disruption [6].

In contrast, the behavior of CDs in the POPE membrane was markedly different. In this system, the CDs were initially captured by the membrane surface at approximately 20 ns but remained adsorbed on the upper surface and failed to permeate the membrane interior. As a result, only one side of the CD atoms established contact with the phospholipid molecules, which is significantly less than the fully embedded CDs in POPC and DOPE membranes. Over time, the angle between the CDs and the POPE membrane surface decreased to around 10 degrees by 172 ns, and the adsorption persisted until the end of the simulation, demonstrating that the CDs were unable to overcome the energy barrier presented by the densely packed POPE molecules [6].

The critical factor underlying these differences was determined to be the molecular density of the phospholipid membranes. POPE membranes exhibit the highest density of phospholipid molecules per unit area, with an average spacing of 58.8 Å^2^ per lipid, compared to 68.3 Å^2^ for POPC and 63.4 Å^2^ for DOPE. This high density in POPE results in smaller gaps between adjacent lipids, which imposes a significant steric barrier that prevents the CDs from entering the membrane interior. Free energy calculations using the Potential of Mean Force corroborated these findings, revealing a substantially high-energy barrier at the surface of the POPE membrane that hinders permeation, whereas POPC and DOPE membranes exhibited a relatively low surface energy barrier and a free energy minimum located within the interior of the bilayer, consistent with the MD observations. To further validate this mechanism, a computationally low-density POPE membrane (POPE-LD) was constructed. In this system, the CDs were able to fully enter the membrane within 50 ns, confirming that reduced lipid density facilitates CD permeation by providing larger gaps between phospholipid molecules [6].

This study also examined the forces driving the translocation of CDs across membranes. The interactions were dominated by van der Waals forces, which were substantially stronger than electrostatic interactions, establishing van der Waals forces as the principal driving force enabling CDs to enter the bilayer. In the high-density POPE membrane, both van der Waals and electrostatic forces were weaker than in POPC and DOPE systems, insufficient to overcome the steric and energetic barriers, explaining the observed surface adsorption without permeation [6].

Taken together as summarized in Table 1 (ref. [6]), these simulations identify lipid molecular density as a dominant energetic regulator of CD membrane translocation, providing a mechanistic baseline for interpreting experimental observations of CD localization and functionality within lipid bilayers.

#### 4.1.2. Experimental Validation Through Fluorescence-Based Membrane Studies

**Figure 4:**
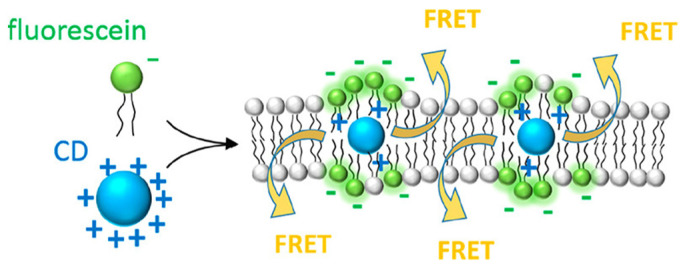
Schematic presentation of the CD/Fluorescein configuration in the DOPC bilayer membrane. Phospholipid molecules that are labelled with negatively charged fluorescein are mixed with DOPC phospholipids (gray) and positively charged blue-emitting CDs to self-assemble into a bilayer membrane. The hydrophobic CDs are located close to the lipid tails between the bilayer leaflets, while the fluorescein molecules are covalently bound to the hydrophilic lipid head groups. Both fluorescein and the CD form a FRET pair that is colocalized within the membrane sheet due to attractive electrostatic interactions. Reprinted with permission from Ref. [19]. Copyright 2019 ACS.

Whereas MD simulations emphasize the role of membrane packing in enabling or preventing CD insertion, fluorescence-based experimental studies provide complementary insight into CD positioning and nanoscale proximity within lipid bilayers. Another study demonstrated fluorescent CDs embedded in a synthetic lipid bilayer, providing a new strategy for optically probing and manipulating membrane-related processes [19]. The work highlights the potential of biocompatible CDs as energy donors in a lipid environment, achieving efficient energy transfer to a dye acceptor across the membrane through electrostatic attraction, as shown in Figure 4.

The system was constructed using a DOPC phospholipid bilayer containing two fluorescent components positioned across opposite leaflets. The donor consisted of hydrophobic, positively charged blue-emitting CDs synthesized from ascorbic acid and hexadecylamine, with particle sizes of approximately 1–1.5 nm and a zeta potential of +60 mV. These CDs were incorporated into the hydrophobic interior of the bilayer. The acceptor was fluorescein (F), a negatively charged dye covalently attached to the phospholipid headgroups at the membrane surface, as shown in Figure 4. The estimated donor–acceptor separation distance was ~3 nm, close enough to facilitate energy transfer. Electrostatic attraction between the cationic CDs and the anionic fluorescein ensured close colocalization and alignment within the membrane, a prerequisite for effective FRET coupling [19].

**Figure 5:**
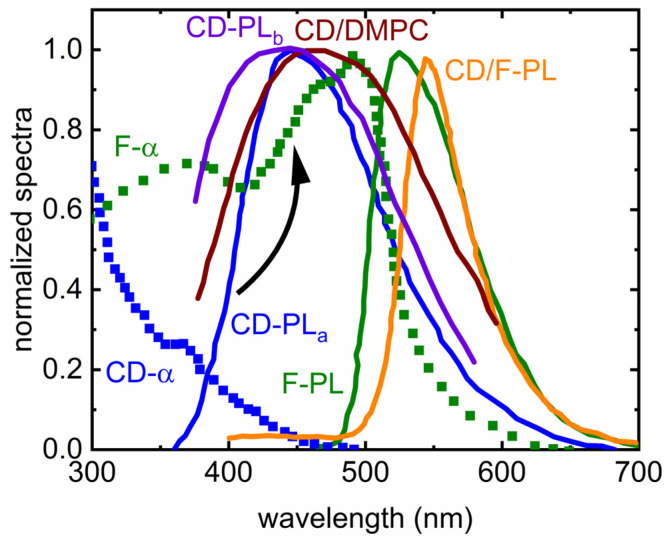
Normalized absorbance (dotted line) and photoluminescence (PL, solid lines) for. CDs (blue), fluorescein (F, green), and CD-fluorescein system (CD/F, orange). PL emission spectra of the CDs (excitation at 350 nm) before (violet) and after (dark red) coupling of the CD to DMPC. The arrow highlights the FRET mechanism. Adapted from references [7,19].

Figure 5 summarizes the key photophysical features that enable efficient energy transfer in CD/fluorescein hybrid systems and illustrates how combining CDs with fluorescein-labeled phospholipids modulates the optical response of both components. Individually, CD-containing vesicles (CD-α and CD-PL) display a characteristic blue emission centered near 450 nm when excited at 360 nm, whereas fluorescein alone (F-PL, F-α) exhibits a broad absorption band spanning ~410–520 nm and a green emission maximum near 530 nm under 450 nm excitation. The substantial spectral overlap between the CD emission and fluorescein absorption, clearly visible by comparing the blue CD curves with the green fluorescein curves in the figure, fulfills a central requirement for FRET. When both fluorophores are co-incorporated within the same lipid environment, the resulting PL spectrum displays the hallmark signatures of donor–acceptor coupling: the CD emission is quenched, and the acceptor fluorescence is strongly enhanced and slightly red-shifted (~525 → 532 nm). This spectral evolution, illustrated by the orange CD/F-PL curve trending upward in the green region, indicates that CDs serve as efficient donors that “feed” excitation energy into fluorescein.

Beyond spectral overlap, the supramolecular arrangement within the membrane also supports FRET. At pH 7.1, fluorescein exists predominantly as a monoanion, while the CDs possess a highly positive surface charge. These opposing charges promote electrostatic attraction, likely drawing the dyes into proximity within the bilayer and helping maintain donor–acceptor distances within the 3–5 nm Förster radius. The figure’s systematic comparison of CD-only, F-only, and CD/F spectra visually reinforces this interpretation: mixtures exhibit the characteristic decrease in donor intensity alongside enhanced acceptor emission. Some of the acceptor enhancement may also arise from electrostatic stabilization effects known to shift fluorescein’s monoanion–dianion equilibrium, producing red-shifted and intensified fluorescence in the presence of cationic species. The absence of spectral features indicative of aggregation, in agreement with microscopy studies showing homogeneous membrane coloration, suggests that the interactions are molecular rather than domain-forming. Collectively, the spectral trends in Figure 5 highlight how combining CDs with fluorescein within a lipid bilayer creates a favorable nanoenvironment for both electrostatic association and efficient energy transfer, providing a tunable optical platform with implications for membrane-based sensing, imaging, and nanoscale energy transport.

Importantly, these fluorescence studies experimentally corroborate computational predictions by confirming that CDs embedded within permissive membranes can maintain stable nanoscale positioning and participate in functional interactions without inducing large-scale bilayer disruption as summarized in Table 1 (ref. [19]).

#### 4.1.3. CDs as Intrinsic Probes of Membrane Dynamics

Beyond acting as membrane-active nanoparticles, CDs have also been engineered as intrinsic reporters of bilayer dynamics, enabling direct correlation between CD-membrane interactions and changes in membrane fluidity, rigidity, and molecular diffusion. A study introduces and validates a novel molecular probe, a fluorescent CD covalently attached to a phospholipid (CD-DMPC phospholipid conjugate), as a highly adaptable tool for studying lipid-bilayer dynamics [7].

The fluorescence emission spectra (350 nm excitation) in Figure 5 reveal an experimentally significant shift in the emission peak of the CDs coupled to DMPC (dark red color) in comparison with the corresponding peak of the unbound CDs (purple color). This probe overcomes key limitations of traditional membrane fluorescent dyes by offering strong photostability, broad excitation/emission ranges, and efficient incorporation into diverse membrane systems, including solid-supported bilayers, small and giant vesicles, and even live cell membranes. Its unique sensitivity to the physicochemical properties of its local environment makes it well-suited for a wide set of biophysical methods. Using fluorescence anisotropy, fluorescence lifetimes, FRET, and FRAP, the study demonstrates that the probe can report subtle changes in bilayer fluidity, lipid mobility, and molecular diffusion with high resolution. These capabilities are particularly important because membrane dynamics underpin essential cellular processes, yet existing probes have been limited by poor insertion efficiency, photobleaching, or restricted spectral behavior [7].

**Figure 6:**
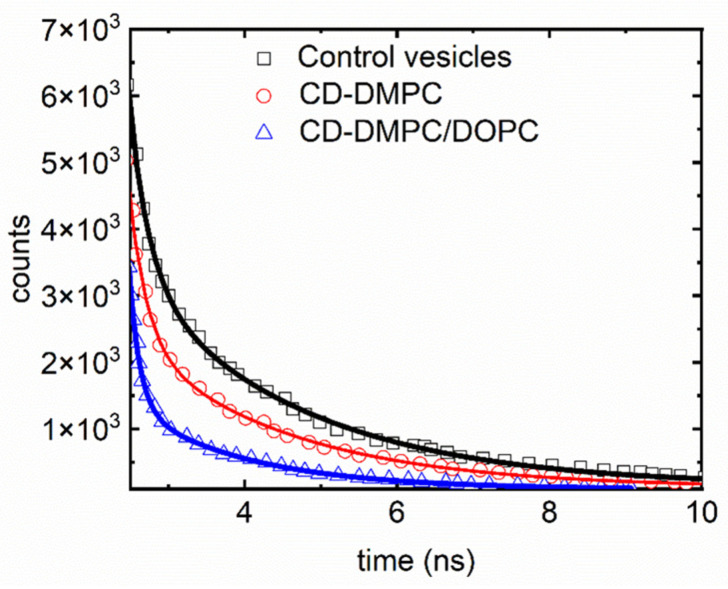
Fluorescence lifetime measurements (symbols) with corresponding exponential fits (lines) for control vesicles (DOPC) before adding membrane-active compounds (black), CD-DMPC embedded in SUVs composed of DOPC and cholesterol (red), and after adding PMB to the CD-DMPC/DOPC SUVs (blue). The fits show sensitivity to the composition by changing time scales drastically in the three variants. Adapted from reference [7].

Fluorescence lifetime data shown in Figure 6 further highlight the probe’s remarkable sensitivity to bilayer composition and molecular perturbation. As shown in the plotted measurements and exponential fits, the control SUVs containing CD-DMPC exhibit an intermediate fluorescence lifetime, while incorporation of 30% cholesterol produces a noticeably shorter lifetime, consistent with cholesterol’s well-known capacity to rigidify membranes and restrict local molecular motions. The most dramatic change appears after the addition of polymyxin-B (PMB); fluorescence decays accelerate substantially, indicating a strong reduction in lifetime and reflecting enhanced bilayer rigidity and disrupted lipid mobility caused by PMB insertion. These trends are entirely consistent with the findings that PMB induces significant bilayer reorganization, while cholesterol produces more moderate but still measurable constraints on lipid dynamics. Figure 6, therefore, reinforces the study’s conclusion that fluorescence lifetime analysis is a powerful and sensitive metric for detecting molecular interactions within the bilayer, and that the CD-DMPC phospholipid conjugate responds with clear, composition-dependent changes in temporal decay behavior [7].

Building on these measurements, the study further demonstrates the probe’s utility by exploring the effects of several biologically relevant membrane-active molecules: polymyxin-B (a cytolytic peptide), valproic acid (a lipophilic drug), and amyloid-β (a peptide implicated in Alzheimer’s disease). Each compound produced distinct patterns across the biophysical assays. PMB caused the largest increase in fluorescence anisotropy, the strongest decrease in fluorescence lifetime, and substantial alterations in FRET efficiency, corresponding to major reductions in bilayer fluidity and increased lipid order. Valproic acid and amyloid-β produced more moderate but still measurable restrictions in lipid mobility, and in FRAP experiments, amyloid-β notably reduced lateral diffusion coefficients, consistent with membrane-associated aggregation. These results confirm that the CD-DMPC phospholipid conjugate is a highly responsive and informative reporter of membrane dynamics, capable of uncovering the mechanistic effects of drugs, peptides, and other membrane-active species at both molecular and larger spatial scales [7]. Collectively, these results demonstrate that CDs can function not only as interacting nanomaterials but also as sensitive optical reporters that translate membrane perturbations into measurable photophysical signatures as summarized in Table 1 (ref. [7]).

Overall, the studies presented in this section and summarized in Table 1 (refs. [6,7,19]) reveal a coherent mechanistic picture: membrane packing density determines CD access to the bilayer interior; surface chemistry governs interaction strength and localization; and compatible CD-membrane interactions enable both structural integration and functional optical behavior. These principles form the foundation for understanding how CDs engage with biological membranes and inform the rational design of CD-based nanoplatforms discussed in subsequent sections.

#### 4.1.4. Factors Affecting the Interactions of the CDs with the Model Membrane System

The interaction of CDs with biological and model membranes is governed by a complex interplay of physicochemical parameters that collectively determine their structural behavior and biological performance. Among these parameters, surface chemistry plays a pivotal role by dictating the hydrophilic or hydrophobic nature of the CDs and controlling their electrostatic, hydrogen bonding, and van der Waals interactions with lipid molecules. The outcome of these interactions, whether adsorption, partial insertion, or full permeation, depends not only on the surface functionalities of the CDs but also on the intrinsic properties of the membrane, including its fluidity, rigidity, and molecular density. Fluid membranes with loosely packed phospholipids, such as those composed of DOPC, tend to facilitate nanoparticle diffusion and insertion, whereas rigid or densely packed membranes like those formed by POPE restrict penetration and favor surface adsorption. These variations in membrane permeability, adsorption, insertion depth, and diffusion dynamics are crucial for understanding how CDs alter or integrate within bilayer structures. Collectively, the balance between CD surface chemistry, the type of interaction forces involved, and the physical state of the lipid bilayer defines the mechanisms underlying CD-membrane association, stability, and transport, ultimately shaping their functional potential in biomedical applications.

##### Surface Chemistry

Comparative studies consistently demonstrate that CD surface functional groups critically determine the strength, mode, and reversibility of interactions with phospholipid membranes. Recent investigations highlight the critical influence of surface functional groups on CDs in determining their interactions with phospholipid vesicles, a model cell membrane system.

**Figure 7:**
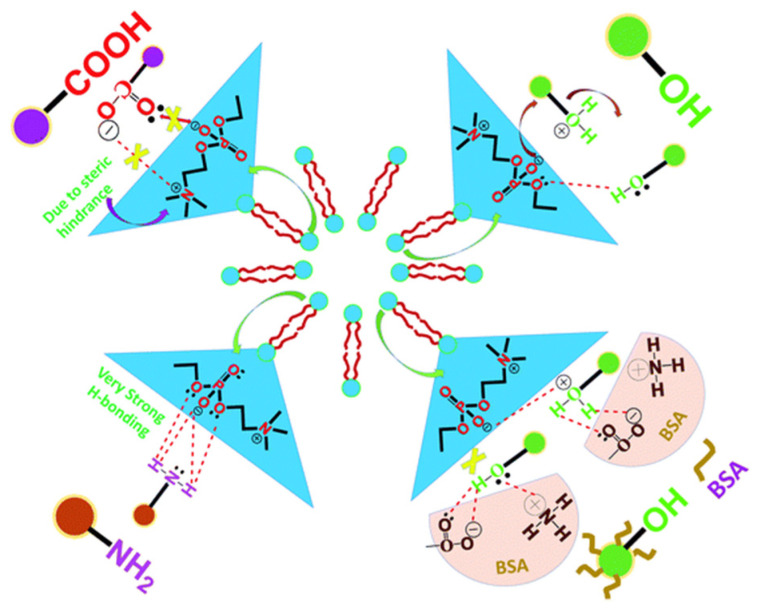
Schematic representation of the probable mechanism of the interaction of CDs with phospholipid vesicles. Reprinted from Ref. [8].

Among various functionalizations, amine-terminated CDs exhibited the strongest affinity for the vesicles, in contrast to carboxylated CDs, hydroxylated CDs, or protein-coated (BSA) counterparts, as shown in Figure 7 and summarized in Table 1 (ref. [8]). This enhanced binding was attributed primarily to hydrogen bonding between the amine groups and the phosphate head groups of the vesicles, as supported by TEM imaging, FTIR, ITC, and QCM measurements [8]. Amine-terminated CDs effectively surround vesicles, quench photoluminescence, and produce a smooth exothermic binding profile, whereas carboxylated CDs, hydroxylated CDs, and BSA-coated CDs show minimal or no significant interaction. The lack of binding for carboxylated CDs is attributed to steric hindrance and limited reactive sites, while hydroxylated CDs and protein-coated CDs fail to form strong interactions due to their functional group chemistry [8].

Importantly, these results establish hydrogen bonding and electrostatic complementarity as dominant contributors to strong CD-membrane association, while steric shielding and surface passivation suppress membrane engagement. The pronounced affinity of amine-functionalized CDs also mirrors biological transport phenomena, in which amine-rich molecules are preferentially trafficked across vesicular and membrane-associated pathways, reinforcing the translational relevance of this surface chemistry for delivery applications [8].

Another study shows that nitrogen-doped CDs (N-doped CDs) can act as drug sensitizers by selectively enhancing membrane permeability in cancer cells, thereby increasing the cytotoxicity of Doxorubicin, an anticancer drug. Slightly positively charged N-doped CDs interact electrostatically with negatively charged liposomal and cellular membranes, with adsorption strongly dependent on ionic strength and temperature [9]. In model liposomal systems, CD-lipid interactions induced moderate changes in zeta potential and a significant reduction in vesicle size, likely due to CDs acting as counterions that modulate membrane curvature. Differential scanning calorimetry indicated that CDs partially embed in the hydrocarbon sublayer without altering the main phase transition, classifying the interaction as Type 3, characterized by increased membrane permeability with minimal structural disruption. The findings of these two studies, as summarized in Table 1 (refs. [8,9]), illustrate that positive charge enables CDs to permeabilize membranes without catastrophic bilayer damage. This balance is critical for therapeutic delivery.

This increased permeability resulted in a concentration-dependent enhancement of doxorubicin release, with release rates and mechanisms indicating a combination of diffusion-controlled and relaxation-controlled processes [9]. Cellular studies corroborated these findings: pre-treatment with N-doped CDs selectively increased doxorubicin uptake and cytotoxicity in breast cancer cell lines, while non-cancerous human dermal fibroblasts remained largely unaffected. This selectivity arises from the higher negative surface charge of cancer cells, promoting stronger electrostatic interactions and localized membrane permeabilization [9].

MD simulations have provided valuable insights into how the hydrophilic and hydrophobic surface properties of nanoparticles (NPs) govern their interactions with biological membranes. Using a DPPC bilayer as a model membrane, this computational investigation revealed that the degree of surface hydrophobicity critically determines whether a nanoparticle embeds into the membrane or remains adsorbed at its surface [13]. These computational findings provide a mechanistic bridge between experimental observations of surface-functionalized CDs and their membrane localization behavior.

**Figure 8:**
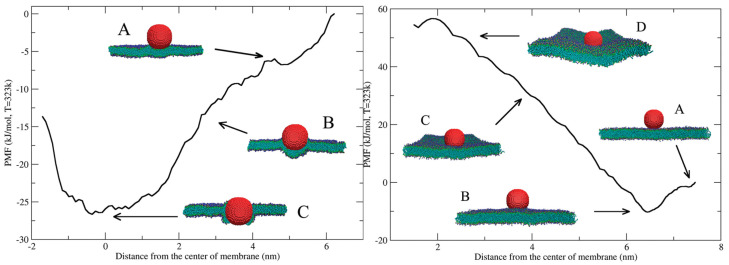
PMF of the hydrophobic NP (**left**) and semihydrophilic NP (**right**) as a function of its distance from the midplane of the DPPC bilayer. Inset images (A–C and A–D) are snapshots corresponding to the sampling positions pointed by arrows. Reprinted with permission from Ref. [13]. Copyright 2008 ACS.

The simulations showed that highly hydrophobic NPs readily insert into the hydrophobic core of the lipid bilayer, as shown in Figure 8 (left). The NPs interact favorably with the fatty acid tails of the phospholipids, leading to partial or complete embedding within the membrane interior. In contrast, semihydrophilic NPs, those with a mixed surface composition, tend to remain adsorbed on the outer surface of the membrane rather than penetrating it, as shown in Figure 8 (right). This adsorption occurs because the balance between hydrophilic and hydrophobic surface sites prevents the particle from overcoming the energetic barrier associated with full bilayer penetration [13].

To further understand these behaviors, free energy profiles were computed for both hydrophobic and semihydrophilic systems. The results demonstrated a clear distinction in the energy landscapes: hydrophobic NPs experience a thermodynamically favorable insertion process, while semihydrophilic NPs encounter a significant energy barrier during membrane wrapping or endocytosis-like events. This barrier indicates that the internalization of partially hydrophilic particles is an energy-dependent process, suggesting that spontaneous embedding is unlikely without additional driving forces such as membrane curvature or external energy input [13].

This study also examined the dynamic deformation of the lipid membrane during NP interaction. The simulations revealed that membrane deformation induced by NPs is localized and short-range, involving transient rearrangements of lipid molecules around the site of contact. These local deformations, driven by changes in lipid packing and molecular orientation, are central to understanding how NPs perturb membrane integrity and structure [13].

These trends rationalize why CDs with intermediate surface polarity often exhibit stable surface association or partial insertion, while highly hydrophobic variants embed deeply within lipid bilayers, as summarized in Table 1 (ref. [13]). Collectively, these studies demonstrate that CD surface chemistry defines a continuum of membrane interactions, ranging from weak adsorption to deep bilayer insertion, with direct implications for permeability modulation, optical behavior, and biological response.

An important biosafety study examined the effects of surface-functionalized CDs on zebrafish embryos, focusing specifically on amino-functionalized CDs synthesized from p-phenylenediamine via a one-step hydrothermal method [74]. Using zebrafish embryos as a dynamic developmental model, the study evaluated both developmental toxicity and the underlying molecular mechanisms. Exposure to amino-functionalized CDs resulted in pronounced, dose-dependent developmental defects, demonstrating a clear correlation between CD concentration and embryotoxicity. Oxidative stress emerged as the central mechanism driving this toxicity, with surface amino groups disrupting redox homeostasis and triggering oxidative damage.

This in vivo evidence reinforces the dual role of surface amine groups: while they enhance membrane interaction and delivery efficiency, they can also amplify oxidative stress pathways when not carefully controlled. Integrating biochemical, metabolomic, and phenotypic evidence, the study proposed a unified model in which amino-functionalized CD surfaces initiate oxidative lipid perturbations that compromise cellular membranes and metabolic pathways, ultimately leading to widespread developmental abnormalities and high malformation rates [74].

##### Isomerism of CDs

**Figure 9:**
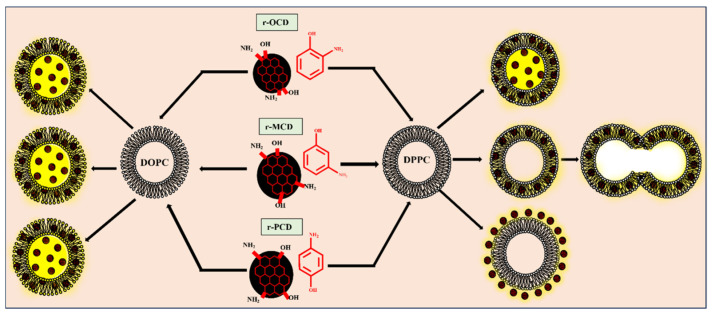
Schematic representation of the interaction between different phase lipid membranes (DPPC/DOPC) and the three isomeric r-CDs, highlighting their phase-dependent interactions. Reprinted with permission from Ref. [14]. Copyright 2025 ACS.

Beyond surface functional groups, subtle variations in CD molecular architecture arising from isomeric precursors introduce another layer of control over CD–membrane interactions. The structural configuration and physicochemical properties of reduced CDs (r-CDs) critically dictate their interactions with lipid membranes, affecting localization, membrane disruption, and suitability for biomedical applications.

r-CDs synthesized from ortho-, meta-, and para-aminophenol isomers (oAMP, mAMP, pAMP) and subsequently reduced with sodium borohydride exhibit distinct hydrophobicity, structural order, and functional group composition, which govern their membrane behavior [14]. The three r-CDs display a hydrophobicity gradient: r-OCD (oAMP-derived) is most hydrophobic, r-MCD (mAMP-derived) is moderately hydrophobic, and r-PCD (pAMP-derived) is most hydrophilic. Spectroscopic and imaging analyses corroborate these trends, showing r-PCD has the highest abundance of hydrophilic oxygen-containing groups, while r-MCD is structurally more ordered with fewer defects, as shown in Figure 9 [14].

Membrane fluidity strongly influences r-CD behavior [14]. In rigid DPPC membranes (gel phase), r-OCD penetrates deeply into the bilayer, stabilizing the membrane via intramolecular hydrogen bonding. r-MCD inserts partially and promotes vesicle fusion through intermolecular hydrogen bonding with lipid headgroups, whereas r-PCD remains confined to the membrane periphery, interacting primarily via electrostatic forces without inducing fusion. In contrast, the fluid DOPC membranes (liquid-crystalline phase) allow complete insertion of all three r-CDs, reducing fusion events and enhancing bilayer integration [14]. These observations, as summarized in Table 1 (ref. [14]), highlight that CD isomerism modulates membrane interactions not in isolation, but in concert with membrane phase state, reinforcing the importance of matching CD structure to target membrane properties.

In another study, the interactions between photoluminescent CDs and zwitterionic lipid vesicles emphasize the critical role of the isomeric precursor used in CD synthesis. CDs were derived from three different isomeric forms of phenylenediamine: ortho-phenylenediamine (oPDA), meta-phenylenediamine (mPDA), and para-phenylenediamine (pPDA). The study demonstrated that the interaction behavior of these CDs with lipid vesicles is highly dependent on the structural and physicochemical properties imparted by the specific precursor. The findings reveal that the precursor type dictates not only the localization of the CDs on or within the vesicles but also the resulting structural integrity of the vesicles, the nature of the interaction, and the potential implications for their biomedical use [20].

**Figure 10:**
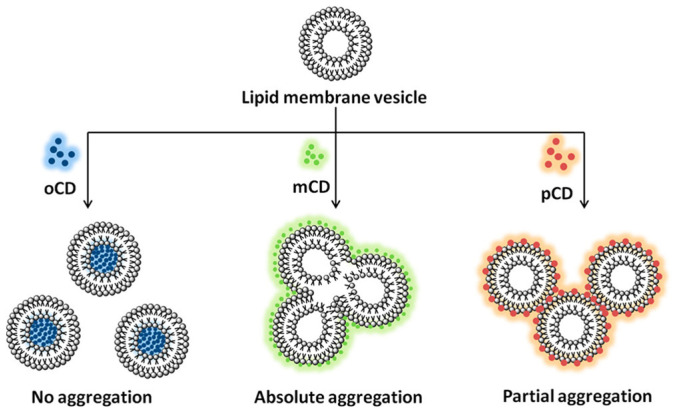
Schematic presentation of the interaction of CDs derived from different isomeric carbon precursors. Reprinted with permission from Ref. [20]. Copyright 2020 ACS.

As shown in Figure 10, Ortho CDs (oCDs) were found to exhibit highly favorable interactions with the lipid vesicles, allowing the vesicles to retain their morphology after exposure. Microscopic imaging confirmed that oCDs are selectively embedded within the lipid bilayer, integrating seamlessly into the vesicular structure. This selective embedding indicates that oCDs interact with the hydrophobic core of the bilayer in a manner that maintains membrane stability, making them particularly suitable for imaging applications where preservation of vesicle morphology is critical. In contrast, meta CDs (mCDs) and para CDs (pCDs) displayed a markedly different behavior, as shown in Figure 10. Rather than embedding within the vesicle bilayer, these CDs localized predominantly at the interfacial region of the vesicles. Their presence at the interface was observed to induce aggregation among the vesicles, suggesting that the interactions of mCDs and pCDs with the vesicular surface are less compatible with the maintenance of vesicle integrity. The interfacial positioning of these CDs appears to facilitate cross-linking between vesicles, potentially destabilizing the membrane and altering the physical arrangement of vesicle populations, as summarized in Table 1 [20].

When considered alongside the r-CD study, these results reveal a consistent isomer-dependent trend: ortho-derived CDs preferentially embed within lipid bilayers, while meta- and para-derived variants favor interfacial localization that can destabilize vesicle assemblies, as summarized in Table 1 (refs. [14,20]). The observed differences in interaction behavior between the three CD types can be explained by several interrelated factors. The precise location of the CDs on the lipid vesicles, embedded within the bilayer for oCDs versus surface-bound at the interface for mCDs and pCDs, plays a pivotal role in determining whether the vesicles maintain their structure or aggregate. Electrostatic attractions between the charged or polar groups on the CDs and the zwitterionic headgroups of the lipid molecules further govern the interaction, with interfacially located CDs exhibiting stronger surface adhesion that promotes vesicle cross-linking. Additionally, the hydration characteristics and surface chemistry arising from the different isomeric precursors contribute to the distinct interaction profiles. Variations in hydration patterns and functional group distribution influence solubility, surface reactivity, and the propensity of the CDs to either integrate within the hydrophobic core or remain at the polar interface. Consequently, the combination of electrostatic effects, hydration features, and steric considerations explains why oCDs embed within vesicles, whereas mCDs and pCDs predominantly remain at the interface, causing aggregation [20]. Together, these studies establish CD isomerism as a structural design parameter that governs membrane compatibility, stability, and functional integration.

In summary, variations in CD surface chemistry and isomeric structure exert systematic and predictable effects on CD-membrane interactions. Surface functional groups dictate interaction strength and permeability modulation, while isomerism controls insertion depth, aggregation propensity, and membrane stability. When integrated with membrane phase behavior, these parameters provide a coherent framework for rationally designing CDs with tailored membrane engagement and minimized toxicity.

### 4.2. CD-Model Membrane Hybrid System

Building on the mechanistic understanding of CD-membrane interactions discussed above, hybrid CD-model membrane systems translate these interaction principles into functional nanoplatforms that combine structural stability, optical traceability, and therapeutic utility. The studies in this section collectively demonstrate how controlled CD incorporation into lipid assemblies enables multifunctional behavior that extends beyond passive membrane interaction.

These hybrid systems, particularly CD-liposome composites, integrate the photoluminescent and imaging properties of CDs with the biocompatibility and encapsulation capabilities of lipid vesicles, resulting in multifunctional nanoplatforms suitable for diagnostics, drug delivery, and theranostics. These CD-model membrane hybrids exhibit well-defined morphologies, enhanced fluorescence properties, and strong interfacial interactions between CDs and lipid bilayers. The incorporation of therapeutic agents (e.g., doxorubicin) further extends their functionality, enabling real-time fluorescence monitoring, pH-responsive behavior, and efficient tumor targeting. Collectively, CD-model membrane hybrid systems represent a promising biomimetic platform that unites physicochemical stability, tunable optical features, and biomedical applicability within a single nanoplatform.

#### 4.2.1. CD-Liposome Hybrids for Imaging-Guided Cancer Theranostics

One prominent class of hybrid systems exploits liposomal confinement to enhance CD optical performance while simultaneously enabling targeted drug delivery and in vivo imaging. The development of Bi-doped CD-liposome hybrid systems represents a significant advancement in the design of multifunctional nanoplatforms for cancer theranosis, integrating both diagnostic and therapeutic functionalities within a single system.

**Figure 11:**
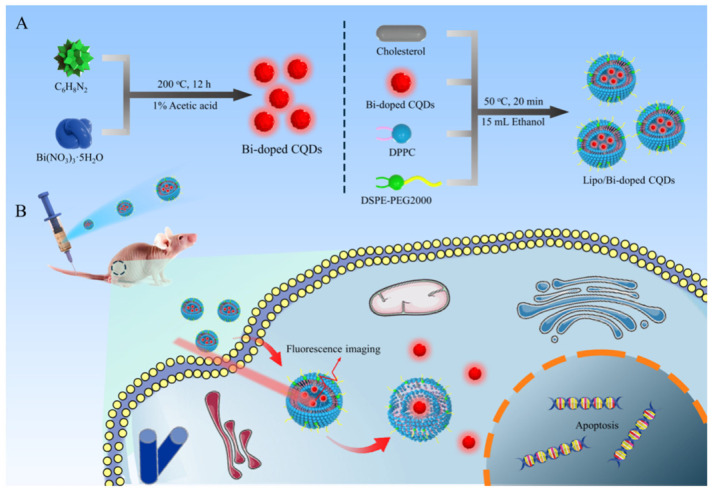
Schematic (**A**) preparation process of Bi-doped CDs and Bi-doped CD-liposome hybrid system, and (**B**) their application in diagnosis and therapy. Reprinted with permission from Ref. [15]. Copyright 2024 Elsevier.

This hybrid nanocomposite combines the superior photoluminescent and imaging properties of Bi-doped CDs with the biocompatibility and drug-carrying capacity of liposomes, enabling simultaneous tumor imaging, targeted delivery, and monitoring of therapeutic efficacy [15]. As shown in Figure 11, the hybrid system was synthesized through a one-step thin-film hydration method, using Bi-doped CDs, prepared by solvothermal polymerization of bismuth nitrate and *o*-phenylenediamine, as the core functional component. Characterization revealed the formation of spherical nanostructures, with Bi-doped CDs averaging 2.7 nm in size, and the final Bi-doped CD-liposome hybrids measuring approximately 78.7 nm, which is smaller than the pristine liposomes (118 nm), confirming successful incorporation and structural modification.

Transmission electron microscopy showed clear lattice fringes corresponding to graphitic carbon planes, while thermogravimetric analysis indicated a high CD loading efficiency (89 wt%). FT-IR and XPS analyses verified the chemical bonding between Bi-doped CDs and liposomal phospholipids, confirming the presence of Bi-O vibrations and oxygen/nitrogen functional groups responsible for excellent water solubility and surface stability [15].

Optical analysis demonstrated enhanced red fluorescence and superior photophysical properties after liposome encapsulation. The fluorescence quantum yield of the CDs increased fourfold in the hybrid system, while fluorescence lifetime decreased, suggesting electron transfer and strong interaction between CDs and liposomal components. The Bi-doped CD-liposome hybrids displayed excitation-dependent emission, high photostability, and excellent aqueous dispersibility, with negligible fluorescence degradation after prolonged UV exposure or salt treatment. These photophysical changes highlight a recurring feature of CD-liposome hybrids: lipid confinement and interfacial coupling can amplify CD emission while preserving colloidal stability under biologically relevant conditions [15].

The hybrid system exhibited strong pH-dependent fluorescence, with maximum emission observed at pH 5, corresponding to the acidic tumor microenvironment. This property enables the system to act as a self-reporting nanoplatform for real-time monitoring of liposomal degradation and drug release kinetics, an advantage over traditional liposomal carriers that lack intrinsic tracking capabilities. In vitro studies using CT26 colon cancer cells confirmed efficient cellular uptake, intense red fluorescence, and low cytotoxicity, underscoring the biocompatibility of the hybrid [15]. In vivo fluorescence imaging in tumor-bearing mice demonstrated that Bi-doped CD-liposome hybrids accumulated preferentially at tumor sites 12 h post-injection, producing strong red fluorescence, while free Bi-doped CDs showed limited localization. This enhanced tumor retention is attributed to the liposomal shell, which facilitates passive targeting through the enhanced permeability and retention effect. Therapeutically, mice treated with the hybrid system showed marked tumor growth inhibition over 15 days compared to control groups, without evident histopathological abnormalities in major organs, indicating excellent systemic safety [15].

#### 4.2.2. CD-Enhanced Liposomal Drug Delivery Systems

Complementary to imaging-focused hybrids, CD-enhanced liposomal systems have also been designed to modulate drug delivery efficiency while enabling fluorescence-based monitoring. A study introduces a dual-functional nanoplatform, CD-Enhanced Doxorubicin Liposomes, developed for targeted cancer therapy with combined diagnostic and therapeutic potential.

The system integrates N-hydroxyphthalimide-derived CDs (CDs-NHF), known for their intrinsic antitumor and fluorescent properties, with liposomes (LPs), established nanocarriers that enable controlled drug delivery. The liposomal systems were synthesized using a modified reverse-phase evaporation method, enabling efficient entrapment of both hydrophilic and amphiphilic molecules, CDs-NHF and doxorubicin (DOX, an anticancer drug) to form the LPs-CDs-NHF-DOX hybrid platform. The hybrid platform aims to improve drug encapsulation efficiency, therapeutic selectivity, and fluorescence-based monitoring while minimizing systemic toxicity, particularly for breast and lung cancer treatments [18].

**Figure 12:**
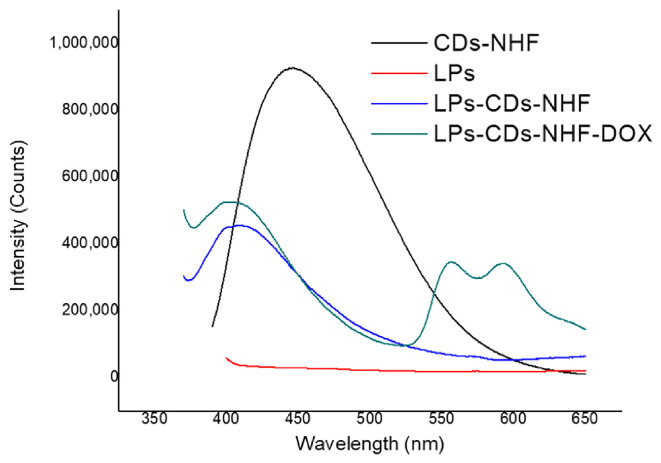
Emission spectra of CDs-NHF (N-hydroxyphthalimide-derived CDs), LPs (liposomes), LPs-CDs-NHF (CDs-NHF loaded in LPs), and LPs-CDs-NHF-DOX (hybrid platform; both CDs-NHF and DOX are loaded in LPs) formulations suspended in H_2_O. Reprinted with permission from Ref. [18]. Copyright 2025 MDPI.

The resulting vesicles were multilamellar (MLVs) and spherical in morphology, with particle sizes ranging from 300 to 1000 nm, as confirmed by Cryo-TEM and Cryo-SEM. These formulations exhibited high encapsulation efficiencies, up to 98.2% for CDs-NHF and 97.7% for DOX, and maintained excellent dispersion stability, as reflected by zeta potential values between −23.67 and −33.12 mV. Optical characterization, as shown in Figure 12, revealed that encapsulation induced changes in the fluorescence behavior of the CDs-NHF, including a blue shift in the emission maximum (from 447 nm to 408 nm) and a reduction in intensity, indicative of partial quenching due to interactions with the lipid bilayer. This inherent fluorescence, combined with DOX’s emission properties, allows dual optical tracking of the hybrid nanocarrier during delivery and release processes [18]. These optical changes underscore how CD-lipid interactions can be leveraged not only for imaging but also to report on carrier integrity and microenvironmental changes during drug transport.

In vitro assays using breast and lung cancer cells demonstrated the strong cytotoxic potential of the developed formulations. LPs-CDs-NHF exhibited greater inhibitory effects on cancer cell viability than free CDs-NHF, while maintaining high biocompatibility toward normal breast epithelial cells. Similarly, DOX-loaded liposomes enhanced cytotoxicity compared to free DOX. The most pronounced effect was observed when LPs-CDs-NHF were combined with soluble DOX, suggesting a synergistic interaction driven by distinct intracellular trafficking mechanisms. In contrast, the co-loaded LPs-CDs-NHF-DOX system showed comparable, but not superior, cytotoxicity to DOX-LPs alone, implying potential competition during endocytosis when both agents are encapsulated together [18]. These results highlight that CD incorporation can enhance therapeutic outcomes, but also that intracellular trafficking and loading strategies critically influence synergy within hybrid systems.

#### 4.2.3. Hybrid Liposome-CD Assemblies as Biomimetic Optical Platforms

Beyond therapeutic applications, CD-liposome hybrids have been developed as biomimetic platforms to probe membrane structure and optical behavior. In another study, a hybrid CD-liposome system was also synthesized and characterized to explore its structural, optical, and functional properties as a biomimetic nanoplatform.

**Figure 13:**
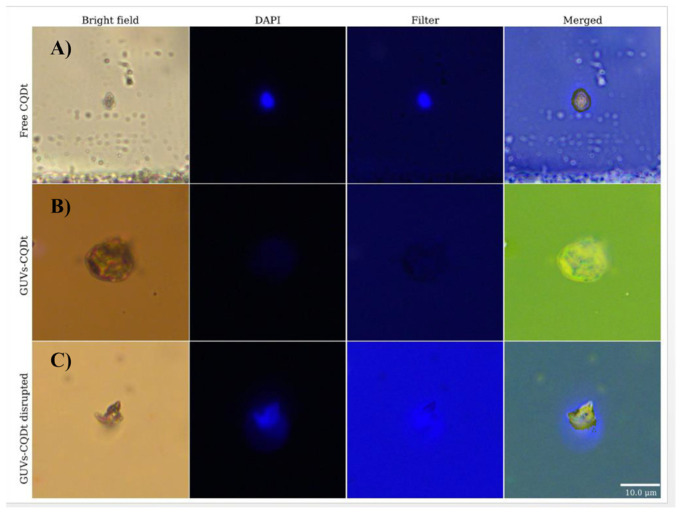
Fluorescence properties of the liposome (GUVs)-CDs hybrid system. Horizontal images applying different filters corresponding to (**A**) Free CDs (CDs are CQDs), (**B**) liposome-CD hybrid system, and (**C**) unstable liposome-CD hybrid system, with CDs outside the lipid. Reprinted from Ref. [17].

Water-soluble CDs exhibiting blue fluorescence were synthesized through a rapid and environmentally friendly electrochemical process conducted in water at room temperature. These CDs were directly encapsulated into liposomes composed of DMPC phospholipids in a single quick step, establishing a sustainable and “green chemistry” approach. The liposomes were initially prepared as LUVs of approximately 100 nm, which later formed hybrid GUVs upon electrode deposition. Electrochemical analyses confirmed the formation and stability of the hybrid CD-liposome structure. The microscopic imaging validated the formation and morphology of the hybrid systems. SEM and optical microscopy showed spherical vesicles, while fluorescence microscopy confirmed CD encapsulation, as shown in Figure 13, displaying three distinct states: free CDs with intense blue emission, well-formed hybrid GUVs with CDs localized inside, and deformed vesicles exhibiting CD leakage. The deformation observed was attributed to strong electrostatic interactions between the charged CDs and lipid bilayers [17].

Surface analysis revealed abundant oxygen-containing functional groups (carboxyl, hydroxyl, amide, carbonyl, and epoxide), contributing to excellent aqueous solubility and facilitating electrostatic interaction with lipid phosphate groups. Results further confirmed interactions between the positively charged CDs and the negatively charged phosphate groups of the DMPC lipids, leading to restricted molecular vibrations and an overall increase in membrane rigidity and stability [17].

These findings mirror trends observed in earlier sections, reinforcing that strong CD-lipid electrostatic interactions can both stabilize and mechanically perturb membranes depending on loading and charge density.

#### 4.2.4. Extending Hybridization Beyond Lipid Membranes

**Figure 14:**
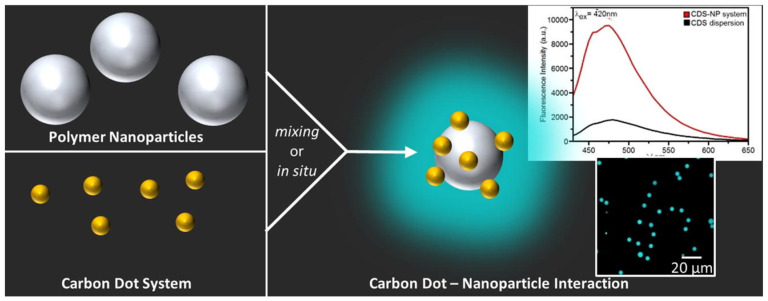
Schematic presentation of the interaction between fluorescent CDs and polymer NPs. Polymer NPs (**top left**) and CDs (**bottom left**) are combined either by simple mixing or through an in situ incorporation process (**middle**), resulting in CD-NP hybrid systems. The resulting hybrid system exhibits enhanced fluorescence, as shown by the emission spectrum (**top right**), where the CD–NP hybrid system displays significantly higher fluorescence intensity compared to the free CDs. Fluorescence microscopy imaging (**bottom right**) further confirms the distribution and optical activity of the CD–NP hybrid assemblies. Reprinted with permission from Ref. [16]. Copyright 2018 Elsevier.

While lipid bilayers provide biologically relevant confinement, analogous interfacial stabilization strategies have been extended to non-membrane systems to enhance CD optical performance. Previous studies in this section primarily focused on CD-membrane hybrid systems. In contrast, this study in Figure 14 extends this concept by introducing a strategy to enhance the photoluminescence properties of CDs through their controlled interaction with the surface of polymer nanoparticles (NPs), rather than with a model membrane system. By combining the optical activity of CDs with the colloidal stability of polymeric carriers, the study demonstrates that both chemical and physical attachment of CDs to polymer NPs significantly improves fluorescence performance and stability, establishing a new pathway for the design of efficient fluorescent nanocomposites for bioimaging and biosensing applications [16].

Two complementary synthetic approaches were developed to construct the CD-NP hybrid systems, employing chitosan, 1,2-ethylenediamine (EDA), and acetic acid as precursors for the CDs, and carboxyl-functionalized polystyrene (PS–COOH) NPs as the support material. In the first approach (in situ formation), the NP dispersion and CD precursors were simultaneously treated under microwave irradiation, yielding CDs covalently anchored to the NP surface via amide bond formation between amine and carboxyl groups, as shown in Figure 14. FTIR spectra confirmed the presence of amide linkages, while pH stability tests indicated that the fluorescence remained stable under acidic conditions, verifying strong covalent attachment. Electron microscopy revealed that after the reaction, the initially smooth PS–COOH NP surfaces became rough and were covered with crystalline domains characteristic of graphitic carbon [16]. In the second approach (mixing method), pre-synthesized CDs were physically adsorbed onto PS–COOH NPs through electrostatic interactions. This noncovalent system displayed pH-sensitive fluorescence behavior, showing decreased intensity under acidic conditions due to the weakening of electrostatic attraction and partial desorption of the CDs. Both systems, regardless of the bonding mechanism, produced a substantial enhancement in fluorescence intensity (approximately fivefold compared to free CDs while maintaining a consistent emission peak at 475 nm, as shown in Figure 14. Moreover, fluorescence lifetime measurements revealed a significant prolongation from 0.95 ns for CDs to about 4.8–5.0 ns in the CD–NP systems. This lifetime extension indicates stabilization of the radiative trap states responsible for emission, suggesting that interaction with the polymer surface suppresses nonradiative decay pathways. The comparable enhancements observed in both covalent and electrostatic systems suggest that the fluorescence amplification is driven primarily by interfacial stabilization effects rather than specific chemical bonding [16].

Further characterization confirmed the homogeneity and biocompatibility of the hybrid systems. Flow cytometry demonstrated uniform labeling of larger microspheres (PS–COOH MSs) with CDs, confirming consistent surface coverage. When applied to HeLa cells, the labeled NPs displayed strong intracellular fluorescence and effective cellular uptake without altering cell morphology, indicating low cytotoxicity and good biocompatibility. The covalently bound CDs remained attached during and after cellular internalization, confirming the structural integrity of the hybrid material under biological conditions [16].

Although polymer-based hybrids lack the biomimetic features of lipid systems, their fluorescence enhancement mechanisms parallel those observed in CD-liposome hybrids, suggesting that interfacial confinement is a general strategy for stabilizing CD emissive states.

Collectively, the hybrid systems discussed in this section demonstrate that integrating CDs into membrane-based assemblies transforms CD-membrane interactions into functional advantages, including enhanced fluorescence, environmental responsiveness, improved biodistribution, and therapeutic efficacy. Across imaging, drug delivery, and biomimetic platforms, CD-liposome hybrids consistently outperform free CDs, underscoring the importance of membrane confinement and interfacial coupling in designing next-generation CD-based nanomedicine platforms.

## 5. Conclusions and Future Outlook

This review summarizes current studies examining the interactions between CDs and model membrane systems, as well as the development of hybrid CD-membrane nanostructures. Collectively, these findings reveal that the physicochemical properties of CDs, particularly surface chemistry, synthesis method, functionalization, and particle size, along with the structural and dynamic properties of the lipid membrane, critically determine the mode and strength of CD-membrane interactions. These interactions range from surface adsorption to deep bilayer insertion and are driven primarily by electrostatic, hydrogen bonding, and van der Waals forces. Surface functionalization, especially amine and heteroatom doping, emerges as a key factor influencing not only the optical and electronic behavior of CDs but also their colloidal stability, dispersion, and biocompatibility, providing a rational basis for tailoring CDs for targeted biomedical functions.

Integration of CDs into phospholipid-based carriers such as liposomes has led to the formation of robust hybrid nanostructures that merge the optical advantages of CDs with the biocompatibility, encapsulation capacity, and controlled release features of lipid vesicles. These CD-liposome hybrids demonstrate strong interfacial coupling, enhanced fluorescence, pH-responsive behavior, and improved drug-loading efficiency, establishing them as promising multifunctional platforms for bioimaging, biosensing, drug and gene delivery, and cancer theranostics. Beyond biomedical applications, surface-modified CDs also show potential as fluorescent probes for membrane biophysics, voltage sensing, and real-time tracking of transmembrane processes, expanding their utility into bioelectronic and diagnostic domains.

Despite these advances, the current understanding of CD-membrane interactions remains limited by the complexity of biological interfaces and the variability in CD synthesis and characterization. Future research should focus on establishing unified models that integrate molecular dynamics simulations with experimental data to elucidate the mechanistic pathways of CD insertion, diffusion, and translocation across lipid bilayers. Moreover, systematic studies are needed to correlate CD structure, surface functionality, and photophysical properties with biological responses to ensure reproducibility and biosafety. Expanding research into stimuli-responsive and targeted CD-membrane hybrids, such as those sensitive to pH, temperature, or redox potential, could open new directions for precision drug delivery and real-time therapeutic monitoring.

In summary, advancing this field will require an interdisciplinary approach combining computational modeling, nanomaterial synthesis, and advanced biophysical characterization. The continued exploration of CD-membrane systems promises not only to deepen our fundamental understanding of nanomaterial-biomembrane interactions but also to enable the rational design of next-generation carbon-based nanoplatforms with optimized structural, optical, and biomedical performance for safe and effective use in future nanomedicine.

## Figures and Tables

**Table 1:** Comparative Summary of CDs-Model Membrane Interaction Studies (Section 4.1, including Section Surface Chemistry and Section Isomerism of CDs).

Data from Study [Ref.]	CD Type/Surface Chemistry/Functionalization	Charge Type	Membrane Model/Composition	Technique(s)	Primary Interaction Outcome/Mechanism/Observed Behavior/Functional Implication
[6]	GQDs	Neutral	POPC, DOPE. POPE	MD Simulations	Insertion into loose membranes POPC/DOPE Surface adsorption on dense membranes POPE
[7]	CD-DMPC Phospholipid conjugate	Neutral/zwitterionic	DOPC, DOPC and cholesterol	FRAP, Fluorescence	Membrane rigidity and diffusion, not disruption
[8]	Amine-terminated CDs	Cationic	Phospholipid Vesicles	TEM, FTIR, ITC, QCM, PL	Strong adsorption and quenching via hydrogen bonding and electrostatic attraction
[8]	Carboxylated/hydroxylated CDs	Anionic/Neutral	Phospholipid Vesicles	TEM, FTIR, ITC, QCM, PL	Minimal interaction. Steric shielding suppresses binding
[9]	N-doped CDs	Moderately cationic	Liposomes (and cancer cell membranes)	DSC, zeta potential, DLS	Electrostatic adsorption causes partial insertion into the bilayer and increased membrane permeability to facilitate drug diffusion
[13]	Hydrophobic vs. semihydrophilic NPs (CD analogs)	Neutral	DPPC	MD simulations, PMF	Insertion vs. surface adsorption
[14]	r-OCD (ortho isomer)	Slightly hydrophobic	DPPC/DOPC	Spectroscopy, microscopy	Deep insertion (DPPC), full integration (DOPC), high membrane compatibility
[14]	r-MCD/r-PCD (meta/para isomers)	Less hydrophobic	DPPC/DOPC	Spectroscopy, microscopy	Interfacial localization, fusion (DPPC), aggregation risk
[19]	Hydrophobic amine-rich CDs	Cationic	DOPC and fluorescein-labeled lipids	FRET, Fluorescence	Stable bilayer insertion and nanoscale colocalization
[20]	oCD vs. mCD/pCD (ortho, meta, para isomers)	Variable	Zwitterionic vesicles	Microscopy	Isomer-dependent stability. oCD embeds into the vesicles, m/pCDs cause aggregation

## Data Availability

No new data were created or analyzed in this study. Data sharing is not applicable to this article.

## Abbreviations

The following abbreviations are used in this manuscript:

CDs	Carbon Dots
rCDs	Reduced CDs
GQDs	Graphene Quantum Dots
CQDs	Carbon Quantum Dots
CNDs	Carbon Nanodots
CPDs	Carbonized Polymer Dots
NPs	Nanoparticles
MDs	Molecular Dynamics
BBB	Blood–brain barrier
ROS	Reactive Oxygen Species
PDT	Photodynamic Therapy
PTT	Photothermal Therapy
SUV	Small unilamellar vesicles
LUV	Large unilamellar vesicles
GUV	Giant unilamellar vesicles
MLV	Multilamellar vesicles
MVV	Multivesicular vesicles
POPC	1-palmitoyl-2-oleoyl-sn-glycero-3-phosphocholine
DOPE	1,2-dioleoyl-sn-glycero-3-phosphoethanolamine
POPE	1-palmitoyl-2-oleoyl phosphatidylethanolamine
DPPC	1,2-dipalmitoyl-sn-glycero-3-phosphocholine
DMPC	1,2-dimyristoyl-sn-glycero-3-phosphocholine
DOPC	1,2-dioleoyl-sn-glycero-3-phosphocholine
LPs	Liposomes
DOX	Doxorubicin
PMB	Polymyxin-B
EDA	1,2-ethylene diamine
PS	Polystyrene
NHF	N-hydroxyphthalimide
o-,m-,p-PDA	Ortho-, meta-, para-phenylenediamine
o-,m-,p-AMP	Ortho-, meta-, para-aminophenol
BSA	Bovine Serum Albumin protein
TEM	Transmission Electron Microscopy
SEM	Scanning Electron Microscopy
FRAP	Fluorescence Recovery After Photobleaching
FTIR	Fourier Transform Infrared Spectroscopy
FRET	Foster Resonance Energy Transfer
ITC	Isothermal Titration Calorimetry
QCM	Quartz Crystal Microbalance
PMF	Potential of Mean Force
XPS	X-ray Photoelectron Spectroscopy
PL	Photoluminescence
